# Primary cilia support cartilage regeneration after injury

**DOI:** 10.1038/s41368-023-00223-6

**Published:** 2023-06-02

**Authors:** Dike Tao, Lei Zhang, Yunpeng Ding, Na Tang, Xiaoqiao Xu, Gongchen Li, Pingping Niu, Rui Yue, Xiaogang Wang, Yidong Shen, Yao Sun

**Affiliations:** 1 Department of Implantology, School & Hospital of Stomatology, Tongji University, Shanghai, China; 2 Shanghai Engineering Research Center of Tooth Restoration and Regeneration, Shanghai, China; 3 State Key Laboratory of Cell Biology, Shanghai Institute of Biochemistry and Cell Biology, Center for Excellence in Molecular Cell Science, Chinese Academy of Sciences, Shanghai, China; 4 University of Chinese Academy of Sciences, Beijing, China; 5 Department of Oral and Maxillofacial Surgery, School & Hospital of Stomatology, Tongji University, Shanghai, China; 6 Institute for Regenerative Medicine, Shanghai East Hospital, Frontier Science Center for Stem Cell Research, Shanghai Key Laboratory of Signaling and Disease Research, School of Life Sciences and Technology, Tongji University, Shanghai, China; 7 Shanghai Institute of Stem Cell Research and Clinical Translation, Shanghai, China; 8 Key Laboratory of Big Data-Based Precision Medicine, School of Engineering Medicine, Beihang University, Beijing, China

**Keywords:** Bone development, Cell proliferation

## Abstract

In growing children, growth plate cartilage has limited self-repair ability upon fracture injury always leading to limb growth arrest. Interestingly, one type of fracture injuries within the growth plate achieve amazing self-healing, however, the mechanism is unclear. Using this type of fracture mouse model, we discovered the activation of Hedgehog (Hh) signaling in the injured growth plate, which could activate chondrocytes in growth plate and promote cartilage repair. Primary cilia are the central transduction mediator of Hh signaling. Notably, ciliary Hh-Smo-Gli signaling pathways were enriched in the growth plate during development. Moreover, chondrocytes in resting and proliferating zone were dynamically ciliated during growth plate repair. Furthermore, conditional deletion of the ciliary core gene *Ift140* in cartilage disrupted cilia-mediated Hh signaling in growth plate. More importantly, activating ciliary Hh signaling by Smoothened agonist (SAG) significantly accelerated growth plate repair after injury. In sum, primary cilia mediate Hh signaling induced the activation of stem/progenitor chondrocytes and growth plate repair after fracture injury.

## Introduction

Longitudinal bone growth is sustained by a growth plate (GP), located between the epiphysis and metaphysis at the ends of long bones. GP provides chondrocytes for endochondral ossification. Either injuries or disorders of the growth plate would lead to the shortening of long bones.^1^ Fracture-induced GP injuries are one of the leading causes of skeletal disorders in growing children, and can cause shortened bone growth by arresting GP function.^2,3^ Interestingly, a proportion of growth plate injuries occurring within the growth plate can be recovered,^4^ but the mechanism is unclear. Thus, the mechanism underlying cartilage repair after children’s GP injuries and whether we could find new targets to promote GP repair are essential questions to be answered. The growth plate is comprised of four morphologically distinct zones along with multiple types of chondrocytes at different differentiation stages: resting zone (RZ), proliferating zone (PZ), hypertrophic zone (HZ), and ossification zone (OZ).^5^ Importantly, several recent studies have identified that the resting zone houses mouse skeletal stem cells(mSSCs), essential populations to maintain the development and homeostasis of the skeletal system,^6^ which can be marked by PTHrP^7^ or FoxA2.^8^ Moreover, mSSCs respond to the growth plate injury and participate in growth plate repair.^8^ However, the latest researches are limited to the identification of stem cell population in RZ, the mechanism of how stem cells in the resting zone are activated to contribute to the GP repair remains to be disclosed.

Among the vertebrate development signaling, Hedgehog (Hh) is well known and essential for chondrocytes differentiation and GP maintenance.^9,10^ Importantly, primary cilia are the major transduction mediators of Hh signaling.^11^ Primary cilium, a critical cellular sensory organelle, is enriched with receptors for essential organ developmental signaling pathways, such as IGF, FGF, Hh, and so on.^12^ The primary cilia dysfunctions cause a series of genetic disorders called ciliopathies.^13^ Importantly, a large number of ciliopathies are characterized by bone dysplasia, such as dwarf, thoracostenosis and polydactyly which related with the disruption of chondrocytes functions.^14–16^ Recently, investigations have explored the crucial role of primary cilia in tissue repair or regeneration such as heart,^17^ bone,^18^ muscle^19,20^ and so on. Disrupting cilia structure in bone mesenchymal stromal cells (BMSCs) inhibits Hh signaling transduction and osteogenic differentiation,^21^ bone development^22^ and repair.^23^ Cilia also orchestrate the regenerative response to skeletal muscle injury through the ciliary Hh signaling.^20^ These findings provide insights into ciliary signaling, such as Hh signaling, as a potential therapeutic target to stem cell activation and tissue regeneration.

Recently, we identified that most chondrocytes in the GP resting and proliferating zone are ciliated cells in young mice, suggesting that cilia may participate in cartilage stem/progenitor cell proliferation and long bone growth. This study delineates how cilia function in GP regeneration after injury. It is observed that: both Hh signaling and ciliation of chondrocytes increased in the resting zone during GP repair. Moreover, homeostasis of the growth plate was disrupted by ciliary gene knockout, and GP cartilage regeneration was improved by SAG-induced Hh-ciliary signaling activation. These results reveal that the ciliary Hh signaling of chondrocytes in the growth plate coordinate the cartilage regenerative response to GP injury and provide new insights into other types of growth plate injury.

## Results

### Cartilage regeneration after GP injury

**Fig. 1:**
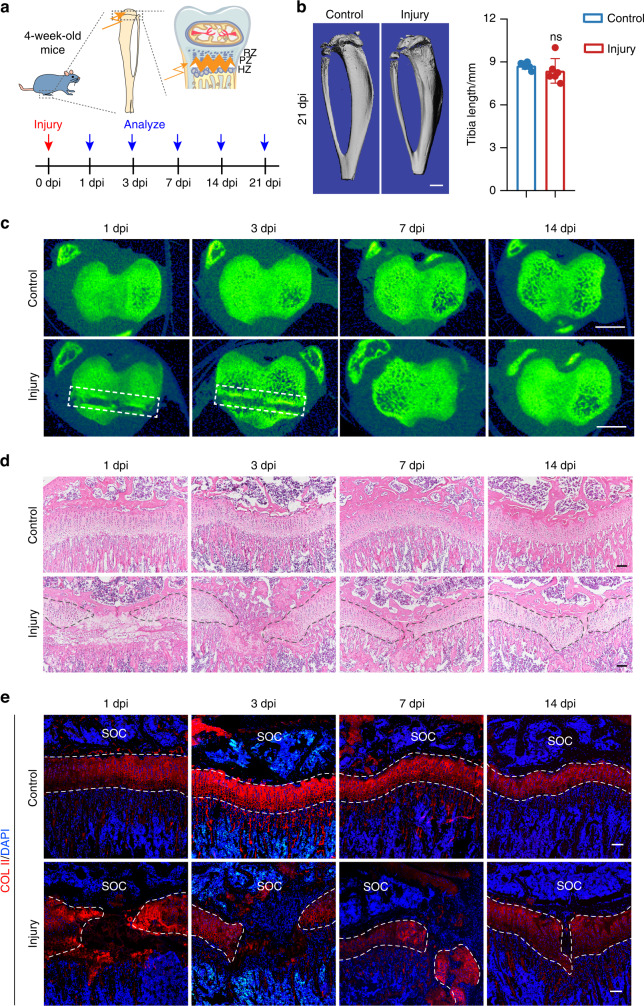
GP cartilage regeneration after injury. **a** Schematics of injury model in the tibia growth plate. **b** Micro-CT images and quantifications of control and operated tibia length at 21 days post-injury (dpi). *n* = 6. Scale bar, 1 mm. **c** Representative micro-CT images of control and operated tibias at 1, 3, 7, and 14 dpi, viewed from the transverse plane. Scale bar, 1 mm. **d** Representative H&E staining of control and operated tibias at 1, 3, 7, and 14 dpi. Scale bars, 100 μm. **e** Immunofluorescence staining for collagen type II (COL II) of control and operated tibias at 1, 3, 7, and 14 days after GP injury. Scale bars, 100 μm. The error bar represents the standard deviation of the mean. ns, no statistical significance

Growth plate injury is one of the most common bone injuries in growing children. To explore its regeneration mechanism after injury, we further inflicted a GP injury involving separation within the growth plate in growing mice.^24^ We constructed an GP injury model to observe the process of GP regeneration (Fig. 1a). Bone length comparison at 21 days after injury revealed that injury within the growth plate had a complete prognosis and did not affect longitudinal bone growth (Fig. 1b). Representative micro-CT images of cross sections between control and operated tibia growth plates revealed that injury site healed within seven days (Fig. 1c) and the injury gap decreased significantly during regeneration. H&E staining was next used at multiple stages to evaluate the GP injury in detail. The central region of the tibia GP being more curved and shorter, the injury caused more damage to this region by a 30 g needle, even the resting zone. The central region was almost destroyed at 1-day post-injury (1 dpi). At 3 dpi, the central gap was closing in. And the GP almost regenerated at 14 dpi, displaying all zones, the injured GP healed with cartilage, not bony structure in other types of GP injury (Fig. 1d). Extracellular matrix remodeling is essential for the repair and regeneration of cartilage.^25,26^ We then observed how GP injury regenerated within 14 days, the expression of type II collagen and proteoglycan increased revealed by anti-collagen type II (COL II) and anti-aggrecan (ACAN) immunofluorescence staining (Fig. 1e, Fig. S1a). These data demonstrate that GP injuries we established can be self-recovered.

Perinatal chondrocytes of growth plate have been demonstrated to form most of the new osteoblasts by one month of age.^27^ Therefore, we analyzed the bone mass underneath the growth plate at 21 dpi, micro-CT scanning showed that the injury group’s bone volume, trabecular number, and thickness significantly decreased compared with the control group (Fig. S1b, c). These data show that GP injuries particularly impact the trabecular bone mass in the primary ossification center.

### GP injury activates Hedgehog signaling of chondrocytes of growth plate

**Fig. 2:**
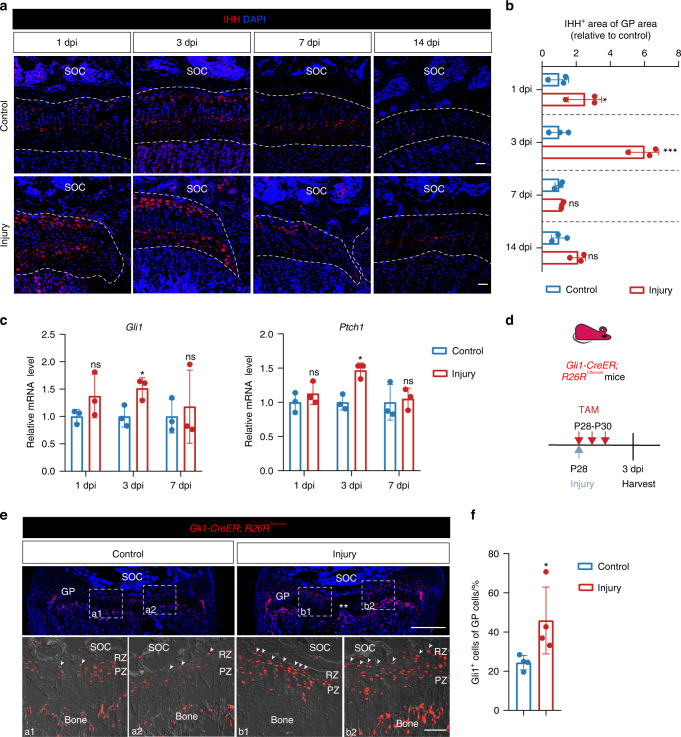
Injured GP presents activated Hedgehog signaling. **a** Immunofluorescence staining for IHH of control and operated tibias at 1, 3, 7, and 14 days after SH1-like surgery. Scale bars, 200 μm. **b** Quantification of IHH^+^ area in the growth plate of control and operated tibias at 1, 3, 7, and 14 days after SH1-like surgery. *n* = 3. **c** mRNA expression level of *Gli1*, *Ptch1* of GP during injury. *n* = 3 per time point. **d** Experimental scheme. Injury in tibia GP of *Gli1-CreER; R26R*^*Tdtomato*^ mice at P28, and pulsed on P28, P29, and P30 with tamoxifen. **e** Confocal imaging of tibia sections from *Gli1-CreER; R26R*^*Tdtomato*^ mice of control and operated-tibias at 3 days post-injury. Insets, area of the growth plate of control and operated-tibias (a1-2, b1-2). GP, growth plate. SOC, secondary ossification center. RZ, Resting Zone. PZ, Proliferating Zone. Scale bars, 500 μm and 100 μm (insets). **f** Quantification of Gli1^+^ (Tomato^+^) cells in GP at 3 dpi. The number of Tomato^+^ cells in the GP was counted in an area from the GP/SOC interface, towards the GP. *n* = 4. The error bar represents the standard deviation of the mean. ns, no statistical significance, *P < 0.05, ***P < 0.001

Given the essential role of the Indian hedgehog (IHH) in chondrocytes proliferation and differentiation, we evaluated the IHH expression during GP regeneration. We found increased IHH at 1 dpi and maintained a high level at the early phase of repair (Fig. 2a, b). IHH is a member of the hedgehog family of secreted ligands. Hh signal transduction activates GLI transcription factors in the presence of Hh signals.^11^ To investigate the activation of Hh signaling during GP injury, we tested the expression level of downstream genes of the pathway, which revealed that the mRNA expression levels of *Gli1* and *Ptch1* were increased at the early phase of GP injury, especially at 3dpi (Fig. 2c). To further determine which cells were activated Hh signaling, *Gli1* reporter mice were obtained by crossing *Gli1-CreER* with *R26R*^*Tdtomato*^ mice and administrated tamoxifen after GP injury immediately (Fig. 2d). Compared with the control group, GP injury group had a higher number of GLI1^+^ cells (Fig. 2e, f). Tomato expression was highly restricted to the growth plate, especially in RZ and the upper part of PZ along the injury site. These observations indicate that GP injury induces IHH expression during the early phase, activating Hh signaling transduction in chondrocytes of resting and proliferating zones.

### Primary cilia mediate Hh signaling transduction in resting and proliferating chondrocytes during GP repair

**Fig. 3:**
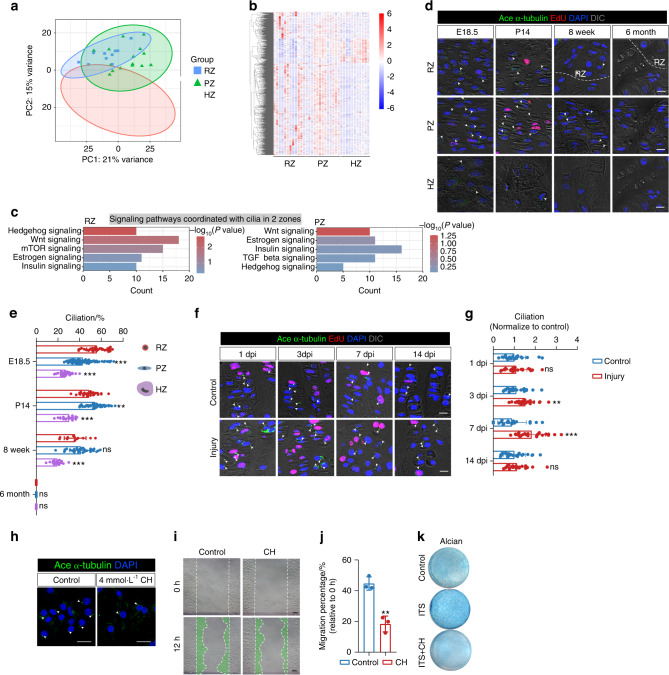
Primary cilia transduct Hh signaling in chondrocytes of RZ and PZ. **a** Analysis of LCM–seq data at P28 between the resting zone, proliferating zone and hypertrophic zone. PCA indicates the different phenotypes among resting, proliferating and hypertrophic zone. RZ, Resting Zone. PZ, Proliferating Zone. HZ, Hypertrophic Zone. **b** Heatmap of significant DEGs among RZ, PZ, and HZ. **c** Enrichment of distinct signaling cascades coordinated with cilia in RZ, PZ. **d** Primary cilia distribution pattern in tibia growth plates of WT mice at E18.5, P14, 8w, 6 m. White arrows indicate primary cilia. Scale bar, 10 μm. **e** Quantification of ciliated cells in RZ/PZ/HZ of the growth plate at E18.5, P14, 8w, 6 m. *n* = 6 mice per age, 5-8 column sections per mice. 2 column sections per mice for RZ at 8w/6 m due to the thinned RZ. **f** Immunofluorescence for cilia (Ace α-tubulin, green) and EdU (red) at the indicated time points after injury of the tibia growth plate of wild-type mice. White arrows indicate primary cilia. Scale bars, 10 μm. **g** Quantification of ciliated cells in the growth plate of control and operated tibias at 1, 3, 7, and 14 days after GP injury. *n* = 3 mice per group per time point, 5-8 column sections per mice. **h** Immunofluorescence for cilia of ATDC5 cells after chloral hydrate (CH) (4 mmol·L^−1^) treatment. White arrows indicate primary cilia. Scale bars, 25 μm. **i** A scratch-wound assay was performed on ATDC5 cells grown to confluency after being treated with CH (4 mM). Images were taken at the start of the experiment (0 h) and 12 hours later (12 h). Scale bars, 100 μm. **j** Quantification of migration by measuring the width of the cell-free zone at the time of the scratch (0 h) and 12 hours after the scratch. *n* = 3. **k** Chondrogenic differentiation of ATDC5 cells after being treated with CH (4 mmol·L^−1^). The error bar represents the standard deviation of the mean. ns, no statistical significance, **P < 0.01, ***P < 0.001

To further investigate the potential mechanism of Hh signaling activation in GP repair, we analyzed the published laser-capture microdissection coupled with RNA sequencing (LCM-seq) data of murine GP at four weeks old,^28^ as the processes of regeneration and development of growth plate cartilage are very similar. Firstly, chondrocytes in the growth plate’s RZ, PZ and HZ showed different gene expression patterns (Fig. 3a, b), with RZ and PZ sharing more similar patterns. At the same time, PZ and HZ were more similar, corroborating what is known about the differentiation trajectory of GP chondrocytes. We further tried to identify differences in the signaling pathways enriched in chondrocytes of each zone, especially Hh signaling, showing a highly enriched in the resting and proliferating zone (Fig. 3c). As vertebrates require primary cilium to interpret Hh signaling and cilia function as signaling nexus for organ development, signaling pathways besides Hh that are coordinated by cilia also highly enriched in RZ and PZ, such as Wnt and Insulin pathways. We next observed the expression of ciliary genes in chondrocytes of various zones. We found these genes to be highly expressed in the resting and proliferating zone, but less expressed in the hypertrophic zone (Fig. S2a). Previous studies identified these ciliary genes tightly related to ciliary structure, signaling and protein trafficking (Fig. S2b). The results of the above LCM-seq data analysis indicated that primary cilia might be closely associated with many essential signaling such as Hh transduction in chondrocytes of GP.

To confirm the distinct distributions of cilia in GP from LCM-seq analysis, we then identified that compared with chondrocytes in the hypertrophic zone, the ciliation of chondrocytes in the resting and proliferating zones was higher (Fig. 3d, e). The proliferation of GP chondrocytes decreased along with the increased cell senescence during development (Fig. S3a–c). We also assessed the IHH expression level in growth plate during bone growth, which showed that IHH expressed robustly especially in the growing stage of bone development (Fig. S3d), and declined after birth. No distinct IHH expression was observed after eight weeks old. These experiments collectively indicated the correlations between primary cilia and GP homeostasis in resting and proliferating chondrocytes.

Then to explore the role of primary cilia in GP injury, we further investigated the ciliation of chondrocytes during GP regeneration. Primary cilia were identified by staining for acetylated α-tubulin, and the ciliation of chondrocytes in injury sites increased during GP repair (Fig. 3f, g). Compared with the tibia GP of the control group, ciliation in RZ and the upper part of PZ increased from 3 dpi and returned to pre-injury levels at 14 dpi. To further investigate the role of cilia in regulating chondrocytes, primary cilia were abrogated by chloral hydrate (CH),^29^ Chloral hydrate treatment decreased ciliation of chondrogenic cells by acetylated α-tubulin staining in vitro (Fig. 3h). The migration and differentiation of chondrogenic cells were reduced by regulating ciliation with chloral hydrate (Fig. 3i–k).

### Ciliary Hh signaling is necessary for the proliferation and differentiation of chondrocytes in GP injury

**Fig. 4:**
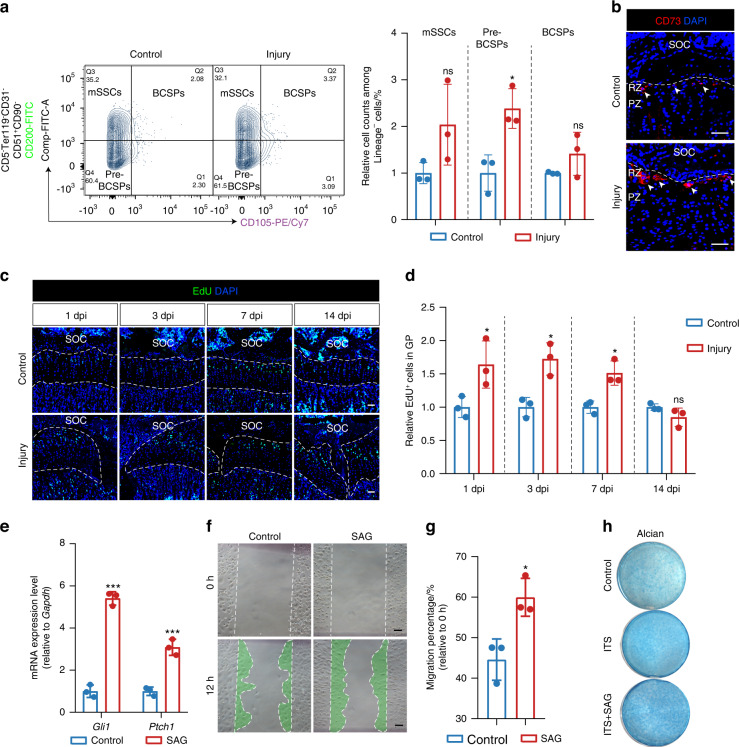
Effects of activated Hh signaling in GP injury and chondrocyte behaviors. **a** Flow cytometry analysis of skeletal stem and progenitor cell-surface-marker of control and operated tibia GP cells at 3 dpi. lineage^-^ cells, CD45^-^Ter119^−^CD31^−^ cells. *n* = 3. **b** Immunofluorescence for stem cell marker CD73 after injury of the tibia growth plate of wild-type mice at 3 days post-injury. White arrows indicate CD73^+^ cells. SOC, secondary ossification center. RZ, Resting Zone. PZ, Proliferating Zone. Scale bars, 50 μm. **c** Tibia growth plates with EdU administration shortly before analysis at 1, 3, 7, and 14 days after GP injury. Scale bars, 200 μm. **d** Imaris quantification of EdU^+^ cells in the growth plate of control and operated tibias at 1, 3, 7, and 14 days after GP injury. *n* = 3. **e** *Gli1* and *Ptch1* mRNA expression were evaluated by real-time PCR after administration of SAG (100 nM) for 24 h in ATDC5 cells. *n* = 3. **f** A scratch-wound assay was performed on ATDC5 cells grown to confluency after being treated with SAG (100 nmol·L^−1^). Images were taken at the start of the experiment (0 h) and 12 hours later (12 h). Scale bars, 100 μm. **g** Quantification of migration by measuring the width of the cell-free zone at the time of the scratch (0 h) and 12 hours after the scratch. *n* = 3. **h** Chondrogenic differentiation of ATDC5 cells after being treated with SAG (10 nmol·L^−1^). The error bar represents the standard deviation of the mean. ns, no statistical significance, *P < 0.05, ***P < 0.001

Stem and progenitor cells in the resting zone maintain GP development.^7^ As IHH regulates chondrocyte proliferation, we next investigated whether activated Hh signaling in GP injury can activate the stem and progenitor cells in vivo and found three important subsets of skeletal stem and progenitor populations:^30^ mouse skeletal stem cells (mSSCs) (CD105^−^CD200^+^), pre-bone, cartilage and stromal progenitors (Pre-BCSPs) (CD105^−^CD200^−^), and bone, cartilage and stromal progenitors (BCSPs) (CD105^+^) were increased after injury at 3 dpi (Fig. 4a). CD73 was identified by previous study as a surface stem cell marker in growth plate.^28^ It was shown that CD73^+^ cells were also increased after injury at 3 dpi (Fig. 4b). It was also observed that the proliferation of chondrocytes enhanced after GP injury by EdU administration (Fig. 4c, d).

Hh signaling transduction via the receptor Patched (PTCH1) and the downstream GPCR Smoothened (SMO) which are highly enriched in the primary cilium.^11^ In order to assess the effect of Hh signaling on cell behavior directly, we intervened the Hh signaling of chondrogenic cells by small-molecule SMO agonists (SAG) in vitro. SAG administration increased the mRNA expression level of *Gli1* and *Ptch1* (Fig. 4e). We then tested the effects of Hh activation on regulating cell migration and differentiation, revealing that SAG promoted migration and differentiation of chondrogenic cells (Fig. 4f–h). Besides, we also inhibited Hh signaling during GP injury by SMO antagonist (LDE225) and found the impaired GP regeneration after administrating LDE225 (Fig. S4a, b), the injury gap was incompletely closed at 7 dpi after ciliary Hh inhibition. These results demonstrate that the activated ciliary Hh signaling via SMO is critical for the proliferation and differentiation of chondrocytes in GP injury, and that inhibiting Hh signaling via SMO can lead to decreased the self-recovered ability of injury within the growth plate during regeneration. All these data indicate that Hh signaling is necessary for GP regeneration.

### Disruption of cilia structure in *Col2*^*+*^ cell impairs Hh transduction and GP development

**Fig. 5:**
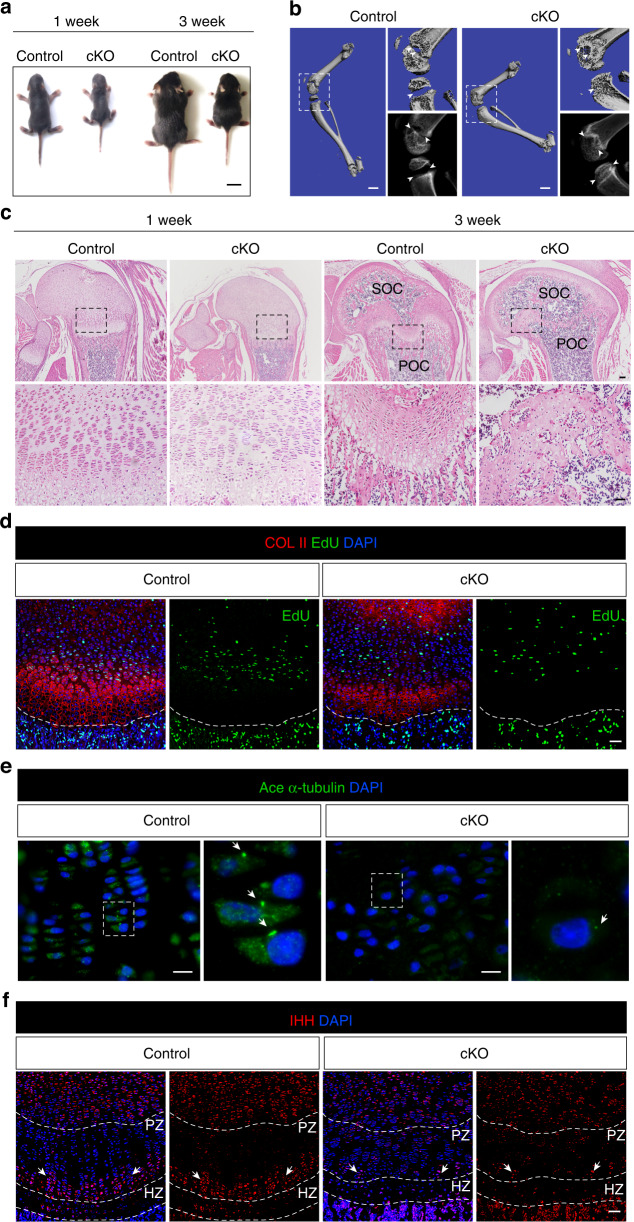
Disruption cilia structure to block Hh signaling in Col2^**+**^ chondrocytes impairs growth plate development. **a** Representative images of *Ift140*^*fl/fl*^ (CTRL) and *Col2-cre; Ift140*^*fl/fl*^ (cKO) mice at indicated ages. Scale bar, 1 cm. **b** Representative micro-CT images showing femur and tibia from *Ift140*^*fl/fl*^ (CTRL) and *Col2-cre; Ift140*^*fl/fl*^ (cKO) mice at indicated ages. Scale bar, 1 mm. **c** Representative H&E staining of distal-femur growth plates showing growth plate morphology from *Ift140*^*fl/fl*^ (CTRL) and *Col2-cre; Ift140*^*fl/fl*^ (cKO) mice at indicated ages. SOC, secondary ossification center. POC, primary ossification center Scale bar, 100 μm. **d** 1-week-old *Ift140*^*fl/fl*^ (CTRL) and *Col2-cre; Ift140*^*fl/fl*^ (cKO) distal-femur growth plates with EdU administration shortly before analysis. Scale bars, 200 μm. **e** Immunofluorescence staining visualizes primary cilia in distal-femur growth plates from one week old mice. Ace α-tubulin stains axoneme. Scale bar, 10 μm. **f** Immunofluorescence staining of IHH in distal-femur growth plates. Arrows indicate the IHH in pre-hypertrophic zone. Scale bar, 200 μm

To further investigate the role of primary cilia in GP chondrocytes in vivo, we specifically deleted *Ift140* in chondrocytes by using *Col2-Cre* mice, which is a core member of IFT A complex that mediating signal transport in primary cilia,^31^ and mutation of *Ift140* could shorten or lose cilia structure. The expression pattern of IFT140 was increased during GP injury, which was consistent with ciliation changes (Fig. S5a). One-week-old *Col2-Cre; Ift140*^*fl/fl*^ mice had short body size and arrested longitudinal development even when three weeks old (Fig. 5a). Moreover, it also had shortened bone length and abnormal metaphyseal morphology (Fig. 5b). Further histological analysis revealed that the *Col2-Cre; Ift140*^*fl/fl*^ mice showed disruption of column arrangement in PZ chondrocytes and decreased height of HZ at one week old (Fig. 5c). At three weeks of age, the growth plate structure in *Col2-Cre; Ift140*^*fl/fl*^ mice was almost completely abolished, while the primary and secondary ossification centers had merged relative to controls. Thus, disruption of primary cilia by deleting *Ift140* in chondrocytes results in reduction of growth plate length and even loss of growth plate structure.

The overall reduction in the growth plate length led us to test the proliferation of chondrocytes within the growth plate by EdU labeling at one week old. It was found that the proliferation of PZ chondrocytes in *Col2-Cre; Ift140*^*fl/fl*^ mice was significantly reduced compared with littermate control (Fig. 5d). IFT140 is one of the important molecules involved in the assembly and function of primary cilia. Immunofluorescent staining was performed to confirm the loss of cilia in *Col2-Cre; Ift140*^*fl/fl*^ mice. As expected, cilia were detected on very few chondrocytes in *Col2-Cre; Ift140*^*fl/fl*^ mice at one week old (Fig. 5e). The expression level of IHH also significantly decreased in *Col2-Cre; Ift140*^*fl/fl*^ mice at one week old (Fig. 5f). These data demonstrate that disruption of primary cilia by deleting *Ift140* decreases the proliferation of GP chondrocytes and impairs Hh transduction in growth plate.

### Ciliary Hh agonist (SAG) promotes GP regeneration in vivo

**Fig. 6:**
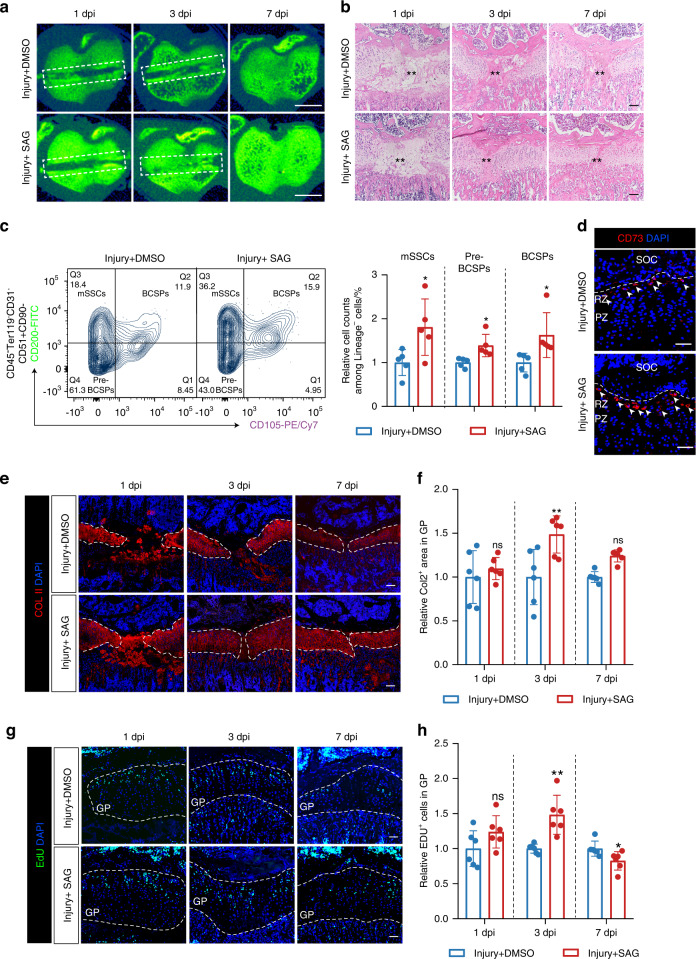
Hh signaling activation promotes GP injury after administrating ciliary SMO agonist. **a** Color maps of representative micro-CT images of operated tibias treated with DMSO or SAG at 1, 3, and 7 dpi. Scale bar, 1 mm. **b** Representative H&E staining of operated tibias treated with DMSO or SAG at 1, 3, and 7 days post-injury. Scale bars, 100 μm. **c** Flow cytometry analysis of skeletal stem and progenitor cell-surface-marker of operated tibias GP cells treated with DMSO or SAG at 3 dpi. lineage^-^ cells, CD45^−^Ter119^-^CD31^−^ cells. *n* = 5. **d** Immunofluorescence for stem cell marker CD73 of operated tibias treated with DMSO or SAG at 3 days post-injury. White arrows indicate CD73^+^ cells. SOC, secondary ossification center. RZ, Resting Zone. PZ, Proliferating Zone. Scale bars, 50 μm. **e** Immunofluorescence staining for collagen II of operated tibias treated with DMSO or SAG, 1, 3, and 7 after GP injury. Scale bars, 100 μm. **f** ImageJ quantification of Col2^+^ area in growth plate at 1, 3, and 7 days after injection of DMSO or SAG. *n* = 6. **g** Operated tibia growth plates with EdU administration shortly before analysis at 1, 3, and 7 days after injection of DMSO or SAG. Scale bars, 200 μm. **h** Imaris quantification of EdU^+^ cells in growth plate at 1, 3, and 7 days after injection of DMSO or SAG. *n* = 6. The error bar represents the standard deviation of the mean. ns, no statistical significance, *P < 0.05, **P < 0.01

Despite the regeneration capacity of GP injury within the growth plate, it took 2–3 weeks to heal completely in mice. To confirm if Hh stimulation could rapidly improve GP regeneration, SAG was administrated in vivo to activate Hh signaling. After applying SAG, GP regeneration was significantly accelerated compared with the control group by micro-CT analysis and HE staining (Fig. 6a, b). The GP injury after SAG administration had healed on the 3rd day, equivalent to that in the control group one the 7th day. The populations of mSSCs, Pre-BCSPs, and BCSPs were all increased after SAG application at 3 dpi (Fig. 6c). And CD73^+^ cells also increased in resting zone after SAG application at 3 dpi (Fig. 6d). The proliferation and differentiation also significantly enhanced in GP injury by activating ciliary Hh signaling (Fig. 6e–h). All these data demonstrate that stimulating Hh signaling via SMO promotes GP regeneration.

**Fig. 7:**
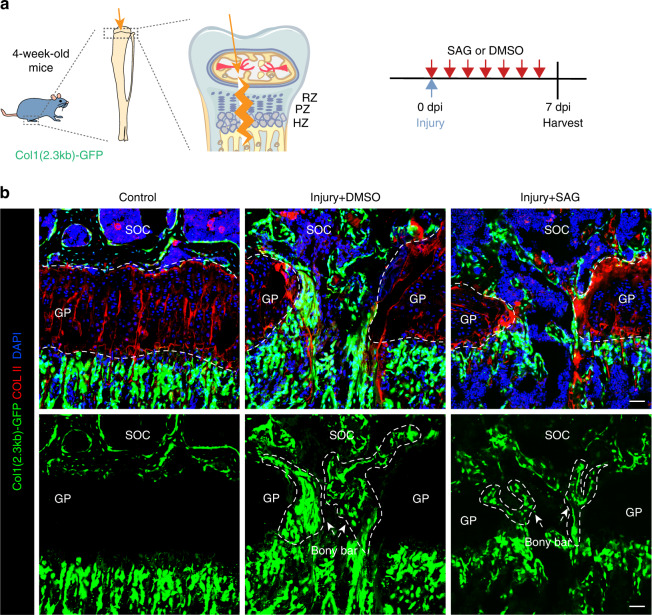
Hh signaling activation inhibits bony structure formation in intractable GP injury. **a** Schematics of intractable injury model in the tibia growth plate. **b** Immunofluorescence for COL II in *Col1a1(2.3* *kb)-GFP* mice of shamed and operated tibias treated with DMSO or SAG at 7 days post-injury. SOC, secondary ossification center. GP, growth plate. Scale bars, 200 μm

As the injury within the growth plate has a good prognosis for repair, then we want to further verify whether activation of ciliary Hh signaling could promote the regeneration of other intractable GP injuries. We established a growth plate micro-perforation injury according to the previous study.^7^ A canal defect from epiphysis to growth plate was made in *Col1a1(2.3* *kb)-GFP* mice to directly observe whether there was bone formation during the GP repair process (Fig. 7a). It was shown that the GP injury has not healed and there was formation of bony bar at the injured gap after 7 days post-injury, the Col1a1(2.3 kb)-GFP^+^ osteoblasts were observed within tibia growth plate at injury sites (Fig. 7b). Although the administration of SAG in this injured model did not completely heal the GP injury, less Col1a1(2.3 kb)-GFP^+^ osteoblasts were observed after application of SAG (Fig. 7b).

## Discussion

**Fig. 8:**
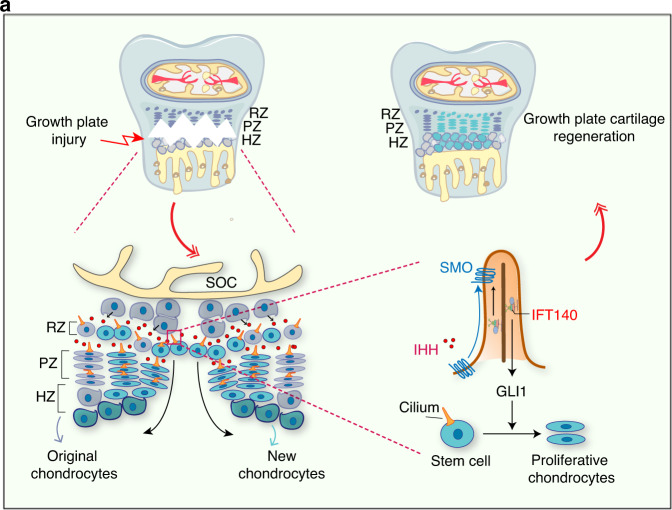
Scheme of primary cilia functions in GP regeneration. Injury within growth plate induces Hedgehog signaling activation in chondrocytes of the growth plate through cilia dependent mechanism, which promotes the proliferation and differentiation of chondrocytes in RZ and PZ of the growth plate

Cartilage damaged by trauma has a limited capacity to regenerate, especially the cartilage injury in growth plate fractures of growing children. Growth plate injuries account for 15–30% of skeletal system injuries in growing children.^32^ How to accelerate GP cartilage healing is an important question that remains to be investigated. Clinically, GP injuries are classified into SH1-SH5 types according to the Salter–Harris (SH) system by different injury positions.^33^ Interestingly, among them, SH1 and SH2 types have the best prognosis, as the cells responsible for regenerate as well as the epiphyseal blood supply remain undisturbed. By constructing the GP injury model within growth plate in growing mice, we observed that the defect of GP cartilage was rapidly repaired. The resting zone is the primary source of stem cells for cartilage regeneration.^34^ However, how stem cells in RZ are activated to form new cartilage after injury remains unclear. Thus, uncovering the underlying mechanisms of stem cell activation during injury repair has an important significance and potential reference for treating SH fractures with the new target molecule and novel strategy. In this study, we uncover an essential role for primary cilia in chondrocytes of growth plate to promote GP regeneration by mediating the Hedgehog signaling pathway (Fig. 8).

Development and homeostasis of the skeletal system involve complicated regulation of signaling pathways such as Wnt,^35^ Hh,^36^ IGF^37^ and so on. Given the master regulation role of cartilage development, Hh signaling should be considered a key candidate for participating in the repair progress of growth plate injury. Hh signaling has been reported to play a crucial role in related tissue regeneration, such as bone^38^ and skeletal muscle.^20^ Here, we demonstrated that IHH was robustly expressed in pre-hypertrophic chondrocytes on both sides of GP injury. The critical question to be answered was how IHH signaling activates chondrocytes differentiation during growth plate repair. *Gli1*, a downstream growth factor of the Hh signaling pathway, has been reported to be a stem cell marker, which could promote stem cell proliferation and differentiation for bone formation.^39^ In our study, we employed *Gli1-CreER; R26R*^*Tdtomato*^ as reporter mice to verify whether Hh activated after injury, and it was shown that the expression level of *Gli1* enhanced in GP after injury, with a notable increase of Gli1^+^ cells in the resting zone in *Gli1-CreER; R26R*^*Tdtomato*^ reporter mice. This observation indicated Hh signaling activation in GP resting and proliferating chondrocytes after injury. Notably, the healing of growth plate injury could be further accelerated by the upregulation of Hh signaling by SAG (SMO agonist) but decreased by Hh inhibition. More importantly, activation of Hh by SAG further inhibited the bony structure formation in other intractable GP injuries, as undesirable formation of bony bridge at the injury site is the direct factor that arrests normal growth of the developing long bone. Therefore, our study showed that Hh signaling activation promotes growth plate repair and it may be a potential target in treatment of GP injury in the future. Although previous publications have reported that Hh activation in the articulate cartilage can cause osteoarthritis (OA) in both mouse models and Human patients,^40,41^ we believe that local or targeting administration system can be further investigated and will be helpful in the further.

It should also be noted that the classical Hh signaling pathway depends on primary cilia for transduction. Primary cilia are cellular protrusions found on most mammalian cells that serve as sensory organelles to sense mixed signals such as growth factors, force, or light.^42^ Primary cilia are essential for both skeletal development and repair. Losing primary cilia core genes function can lead to growth plate disorders and arrest for bone development.^43^ In GP, we identified that the ciliation of chondrocytes highly occurred in RZ. In addition, essential cilia-related signaling pathways such as Hh are enriched in RZ and PZ by using LCM-seq analysis. This observation corroborated the previous finding that primary cilia mainly regulate the differentiation of stem and progenitor cells.^44–46^ Moreover, after the rapid development stage of a long bone, the ciliation of chondrocytes in the GP significantly decreased. Also, loss of ciliation in the aged cell is a cell-intrinsic change that has been identified.^47^ Although accumulating evidence suggests that primary cilia are pivotal in chondrogenesis and growth plate development, little is known about the role of cilia in the regeneration of growth plate injury. In this study, we focused on exploring the role and mechanism of cilia in regulating growth plate regeneration and along this line, we demonstrated that newly formed chondrocytes are dynamically ciliated during GP regeneration. In addition, the ablation of cilia decreased migration and differentiation of chondrogenic cells. Thus, cilia of GP chondrocytes are required to induce the cartilage repair in damaged GP. In addition, to test the role of cilia in cartilage repair, we further blocked the Hh signaling in it by targeting IFT molecules. In the cilium, Intraflagellar Transport (IFT) is required for both the assembly and function maintenance of cilia.^48^ Therefore, to better illustrate how cilia mediate chondrogenic differentiation and cartilage repair, we intervened IFT core molecule to prove the essential role of cilia in the transport of Hh and cartilage repair. IFT-A complex is known to regulate retrograde IFT in cilia, and gene mutation of IFT-A complex members always causes cilia defects and skeletal phenotypes, such as shorter long bone.^49,50^ As the core member of the IFT A complex, mutation of the IFT140 gene has been identified in patients with severe bone dysplasia, short stature and developmental bone malformation,^51^ and these defects are always related to the disruption of chondrocyte differentiation in the growth plate. Our previous investigation has shown that deletion of IFT140 in Osx^+^ cells resulted in less bone mass and abnormal structure of GP.^52^ In this study, we observed that the increased ciliation of chondrocytes accompanied the enhanced expression of IFT140 after GP injury. Here, we demonstrated that disruption of cilia by deleting *Ift140* in chondrocytes with *Col2-Cre* resulted in the loss of cilia-Hh signaling, disorganized growth plate structure, and severe bone development arrest. All these data support the robust correlation of GP repair to IFT-induced ciliogenesis. To further explore the role of primary cilia in GP regeneration after injury, we should have designed to construct injury model by using*Col2-Cre; Ift140*^*fl/fl*^ mice. However, due to the high lethality of 3-week-old *Col2-Cre; Ift140*^*fl/fl*^ mice, it’s unable to construct the injury model with this knockout model to observe the GP regeneration process. Inducible Cre mice will be ideal for investigating this essential process in the future. Keeping on those lines, we activated ciliary signaling in GP by SAG, an activator of ciliary receptor *Smo*, Finally, we demonstrated the underlying mechanism of accelerated GP repair, which validated the role of primary cilia in GP regeneration after injury in a substitution way and provided important information for targeting cilia to repair GP injury in the future.

Overall, this work reveals the critical functions of cilia in coordinating GP regeneration in growing mice long bone. This function is carried out by mediating Hh signaling to active GP chondrocytes. The pharmacological activation of Hh-cilia-Gli1 axis could promote GP defect repair effectively. Meanwhile, our results also reveal a new mechanism by how cilia maintain GP functions and may help identify novel strategies to combat GP injury to rescue more growing children with GP fractures.

## Materials and methods

### Mice

All mice were maintained under C57BL/6J background in a specific pathogen-free (SPF) facility under a 12/12 h day/night illumination cycle. *Gli1-CreER* mice, *R26R*^*Tdtomato*^ mice, and Col1a1(2.3 kb)-GFP mice were used as described previously.^39,53^ All animals were bred according to the National Institutes of Health’s Guide for Care and Use of Laboratory Animals. All animal studies were carried out in accordance with the guidelines of the Institutional Animal Care and Use Committees of Tongji University, and followed all ARRIVE recommendations (Animal Studies: Reporting of In Vivo Experiments) guidelines.

### Growth plate injury model

As described previously, an injury model within the growth plate was established in male wild-type mice.^8^ Briefly, 4-week-old mice were performed under general anesthesia by isoflurane inhalation. Before the operation, the tibias were shaved, and disinfected by iodophor balls. The joint capsule between the femur and tibia was incised and opened with microsurgical scissors. A 30 g needle was passed through the hypertrophic zone of the proximal tibial growth plate. The control mice used in this study are sham-operated. The joint capsule and skin were closed with a 6–0 suture and a 4–0 suture (Jinhuan Medical, China).

### Micro-CT analysis

Dissected tibias were fixed in 4% paraformaldehyde (PFA) for 24–48 h. The tibias analyzed by micro-CT 50 (Scanco Medical, Zurich, Switzerland) at a scan resolution of a 14 μm slice increment with a voltage of 70 kV and a current of 200 μA. One hundred slices of trabecular bone underneath the growth plate were reconstructed for statistical analysis. The parameters of trabecular bone volume/total volume (BV/TV), trabecular bone number (Tb.N), trabecular bone thickness (Tb.Th), and trabecular bone space (Tb.Sp) were quantified according to the standard procedures.

### Histological assessment

For histological analysis, tibias were decalcified in 10% EDTA (pH 7.4) at 4 °C for 3–4 weeks. After dehydration through a series of graded ethanol concentration, specimens were embedded in paraffin and cut into 5-μm-thick sections. Then sections were deparaffinized with xylene and rehydrated in a descending series of ethanol concentrations. Hematoxylin and eosin (H&E) (Sangon Biotech, Shanghai, China) staining was performed for morphological evaluation. The sections were stained with Safranin O/Fast Green (SO/FG) (Solarbio, Beijing, china) for extracellular matrix analysis in growth plate according to the manufacturer’s protocol.

### Immunofluorescence and confocal imaging

For immunofluorescence staining, tibias were embedded in optimal cutting temperature compound (OCT) (Sakura, Taizhou, China) and sectioned at an 8 μm thickness after decalcification. Antigen retrieval was performed using hyaluronidase or 0.1% trypsin; sections were blocked in 5% FBS in PBS-T (0.1% Triton X-100 in PBS), The sections were incubated overnight at 4 °C with anti-type II collagen (COL II) (1:200, Boster Biological Technology, Wuhan, China), anti-aggrecan (ACAN) (1:200, Boster Biological Technology, Wuhan, China), anti-IHH (1:500, Proteintech Group, Wuhan, China), anti-acetylated α-tubulin (1:1 000, Sigma-Aldrich. St Louis, MO), anti-CD73 (1:200, Proteintech Group, Wuhan, China), anti-IFT140 (1:50, Proteintech Group, Wuhan, China) On the second day, the sections were incubated with the appropriate secondary antibody conjugated with fluorophore (1:1 000, Invitrogen, Carlsbad, California). Sections were subsequently stained with DAPI (Sigma-Aldrich. St Louis, MO). All fluorescence microscopy images were acquired using Nikon TI2-E + A1 R confocal microscope (Nikon, Japan) or Eclipse Ni-U microscope (Nikon, Japan). Imaris microscopy Image Analysis Software (Oxford instruments) was employed for counting cell numbers by using spot detection. In brief, DAPI^+^ cell and EdU^+^ cell numbers in growth plate were counted by each channel and then calculate the EdU^+^ cells of growth plate, and methods of quantification of Gli1^+^ cells and ciliation in growth plate were same. ImageJ software (US National Institutes of Health, United States) was used to quantify the fluorescence area of IHH and COL II within growth plate that followed by standard protocol.

### Analysis of LCM-seq data

The counts matrix containing the number of counts for each gene and each sample was obtained from the GEO database (GSE113982).^28^ We thank Phillip T. Newton et al. for sharing the LCM-seq data of growth plate on GEO database. The analysis was performed using R version 4.1.3. Differential expression analysis was performed using the DEseq2 R package. Differentially expressed genes were collected for the heat map by using pheatmap package. Pathway analysis of differentially expressed genes was analyzed by using Kyoto Encyclopedia of Genes and Genomes (KEGG) enrichment analysis. The ggplot2 package was used to visualize the expression prolife of ciliary genes.

### Cell culture

Chondrogenic cell line ATDC5 was cultured as previously described.^54^ Cells were maintained at 37 °C and 5% CO_2_. DMEM (Gibco, Shanghai, China) with 5% FBS containing 1% Insulin-Transferrin-Selenium (ITS) supplement (Gibco, Shanghai, China) and vitamin C were gently added to induce chondrocyte differentiation for fourteen days. 4 mmol·L^−1^ chloral hydrate (Sigma-Aldrich. St Louis, MO) was added to cells to remove primary cilia every two days. 10 nmol·L^−1^ SAG (Selleck, Shanghai, China) was added to cells during chondrogenic differentiation. Cell pellets were fixed with 4% PFA for 15 minutes and stained with Alcian-Blue Staining Solution (Solarbio, Beijing, China) for 30 min to characterize the chondrogenic differentiation.

### Scratch-wound assay

ATDC5 cells were plated into a 24-well culture plate for 24 h. Add medium with 100 mmol·L^−1^ SAG for one day or 4 mmol·L^−1^ chloral hydrate for two days. The culture medium was then removed, and the inoculated cells’ surface was scratched with a 10 μL pipette tip and marked the scratch wound. Then they were gently washed with PBS to remove the floating cells and added culture media with 0.5% FBS. Photograph the scratches at 0 h and 12 h. The distance that the cells migrated to the wounded area during this time was measured by ImageJ software (US National Institutes of Health, United States).

### EdU cell proliferation assay

EdU (5-ethynyl-2’-deoxyuridine, Beyotime, Shanghai, China) dissolved in PBS was administered to mice twice, at six and three hours before euthanization at the indicated days (500 µg for 4 w per injection). BeyoClick™ EdU-594 or 488 (Beyotime, Shanghai, China) was used to detect EdU in the paraffin section.

### Pharmacological manipulation of Hedgehog signaling pathway

For Hedgehog agonist or antagonist experiments, SAG (Selleck, Shanghai, China) or LDE225 (Selleck, Shanghai, China) was reconstituted in DMSO as a stock solution (20 mg·mL^-1^) and kept in -20 °C until use. SAG or LDE225 was diluted in Sunflower oil (Macklin, Shanghai, China) and intraperitoneally administered at 25 µg/g b.w., once a day for seven days. (total seven doses).^7^

### Quantitative real-time PCR assay

Growth plate was isolated under stereoscopic microscope by using microforceps to remove the articular cartilage and secondary ossification centers. The total RNA was isolated from a growth plate or ATDC5 cells with RNAiso Plus reagent (Life, Shanghai, China). cDNA was synthesized using a First Strand cDNA Synthesis Kit (TaKaRa, Beijing, China). Real-time PCR was conducted with a SYBR Green Master Mix (Yeasen, Shanghai, China). The expression levels of mRNAs were normalized to that of the housekeeping gene *Gapdh*. Gene-specific primer sequences are listed in Supplementary Table 1.

### Flow cytometry

Proximal epiphyses of the tibia were manually dislodged, and attached soft tissues and woven bones were carefully removed using forceps. Then isolated the growth plate structure under stereoscopic microscope by using microforceps to remove the articular cartilage and secondary ossification centers. Dissected epiphyses were minced using microsurgical scissors and incubated with 3 mg·mL^-1^ collagenase I and 0.2% collagenase II (Sigma-Aldrich) in Hank’s Balanced Salt Solution (HBSS, Sigma-Aldrich) at 37 °C for 30 min. Cells were mechanically triturated using a vortex and filtered through a 70 μm cell strainer (Corning, NY, USA) into a 15 mL tube on ice to obtain a single-cell suspension. After washing, tissue remnants were incubated with collagenase at 37 °C for another 30 min, and cells were filtered into the same tube. Cells were pelleted and resuspended in FACS buffer (Hank’s buffer, 2%FBS, 5 mmol·L^−1^ EDTA) for flow cytometry. Following standard protocols, the dissociated cells were stained with the following antibodies (1:200, eBioscience, Shanghai, China) to identify the mSSCs, pre-BCSP, BCSP. Allophycocyanin (APC)-conjugated CD31 (390), CD45 (30F-11), Ter119 (TER-119), PE/Cy5-CD90.2 (53-2.1), phycoerythrin (PE)-conjugated CD51 (RMV-7), PE/Cy7-conjugated CD105 (MJ7/18), fluorescein isothiocyanate (FITC)-conjugated CD200 (OX90). Flow cytometry analysis was performed using a four-laser BD LSR Fortessa (Ex. 405/488/561/640 nm, BD, USA) and BD FACSDiva software. Acquired raw data were further analyzed on FlowJo version 10.0.7.

### Senescence-associated β-galactosidase (SA-β-Gal) detection

The SA-β-gal assay was performed according to the manufacturer’s protocol (Beyotime, Shanghai, China). Briefly, tibias were embedded in OCT and sectioned at 8 μm thickness after decalcification. The sections were fixed for 15 min and washed with 1× PBS, and then incubated with staining solution overnight at 37 °C.

### Growth plate micro-perforation surgery model

4-week-old male Col1a1(2.3 kb)-GFP mice were performed anesthesia and disinfection, and the surgery model was established as mentioned previously.^7^ Left tibias were operated, while right tibias were untreated and used as an internal control. All mice were randomly divided into administration of DMSO and SAG group. The joint capsule between the femur and tibia was incised and opened with microsurgical scissors. A hole was made in the intercondylar region of tibias using a 26-gauge needle and then remove a cylindrical area of the growth plat in a stepwise manner by endodontic K-files (#20, 25, 30, 35) (Dentsply, PA, USA).

### Statistics

Statistical data are presented as the mean ± SD. Comparisons between the two groups were analyzed using a two-tailed, unpaired Student’s *t*-test. A one-way ANOVA test was used when the data involved multiple group comparisons. GraphPad PRISM v.8.0.1 was used for statistical analysis. *P* < 0.05 was considered statistically significant.

## Supplementary information

Supplementary figures

Supplementary table
