# Supplementary material for: Primary cilia support cartilage regeneration after injury
Source: Int J Oral Sci. 2023 Jun 2;15:22. doi: 10.1038/s41368-023-00223-6 (PMC10238430; doi:10.1038/s41368-023-00223-6)
Supplement: Supplementary file 1 — Supplementary figures [file 41368_2023_223_MOESM1_ESM.docx]

**
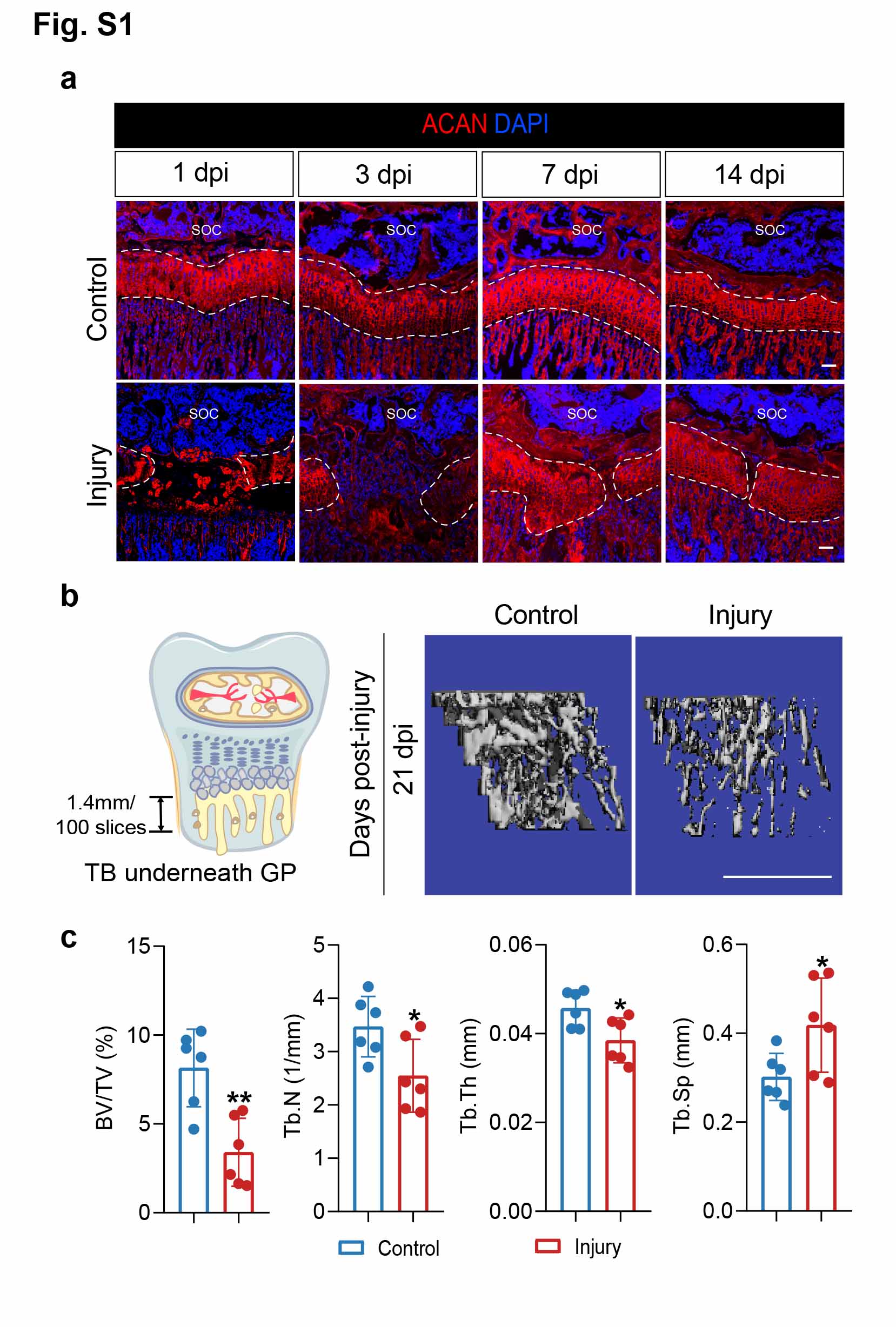
Fig. S1** **GP injury impairs bone remodeling underneath the growth plate. a** Immunofluorescence staining for aggrecan (ACAN) of control and operated tibias at 1, 3, 7, and 14 days after surgery. Scale bars, 100 μm. **b-c** Micro-CT images and quantifications of trabecular bone underneath the control and operated tibias growth plate at 21 days post-injury. TB, trabecular bone. Trabecular bone volume/tissue volume (BV/TV), trabecular number (Tb. N), trabecular thickness (Tb. Th), trabecular bone space (Tb. Sp). n=6. Scale bar, 1mm. The error bar represents the standard deviation of the mean. **P* < 0.05, ***P*< 0.01.


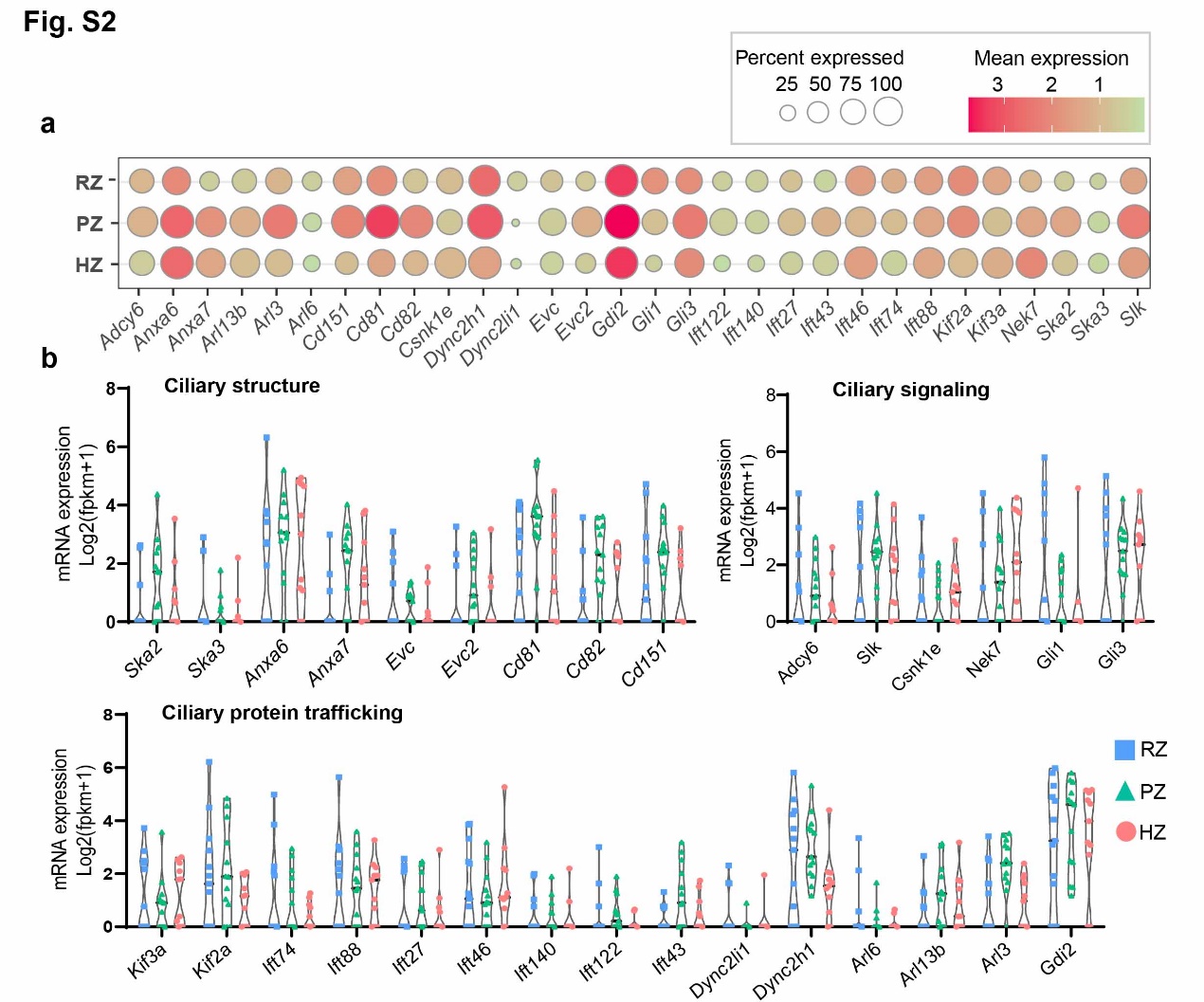
**Fig. S2 Gene expression profile of ciliary genes in different zones of the growth plate. a** Gene expression of ciliary genes in each distinctive zone. **b** Violin plots represent ciliary genes’ expression related to ciliary structure, signaling and protein trafficking in 3 zones.

**
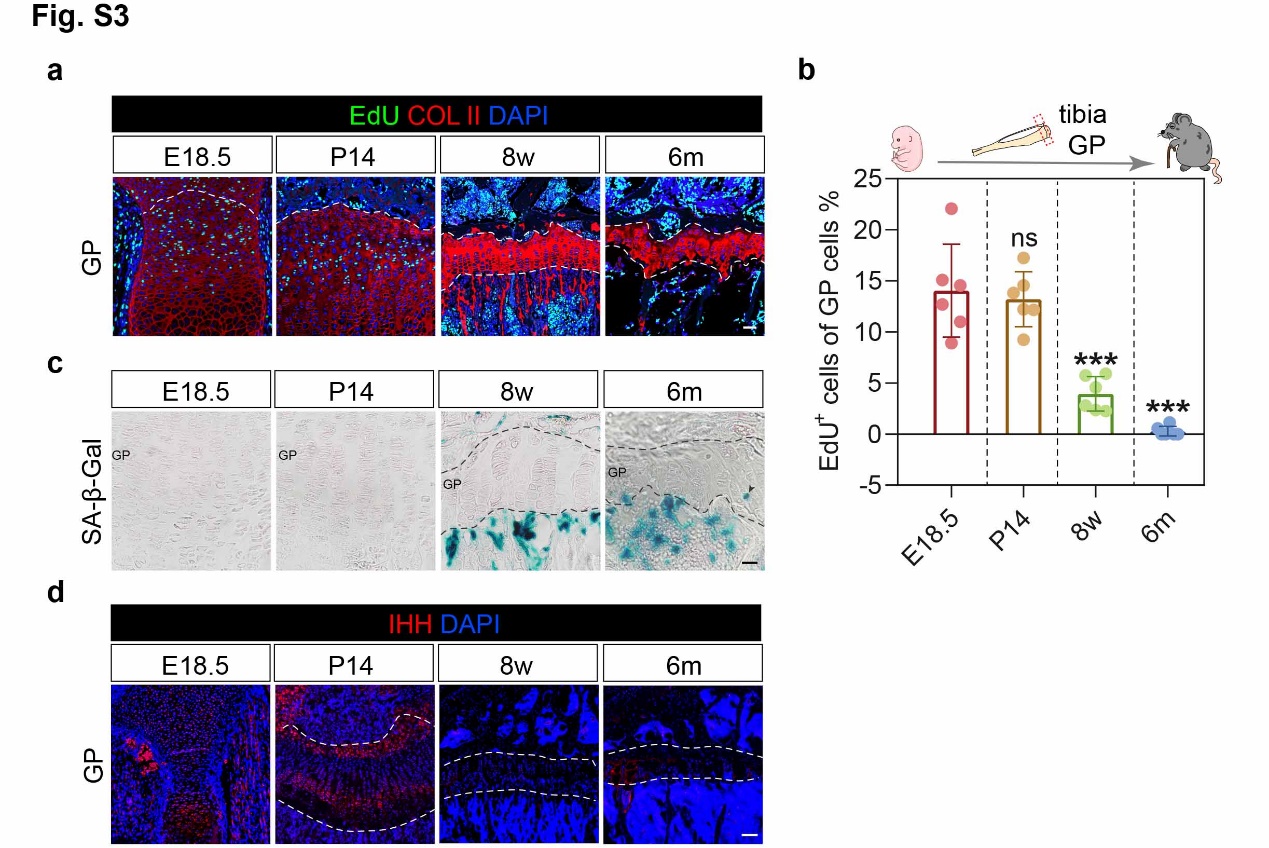
Fig. S3 Proliferation, senescence and IHH expression of chondrocytes in growth plate during bone development. a** Tibia growth plates with EdU administration shortly before analysis at E18.5, P14, 8w, 6m. Scale bars, 200 μm. **b** Quantification of EdU labeling cells in growth plate at E18.5, P14, 8w, 6m. n=6 per age. **c** Senescence-associated beta-galactosidase (SA-β-gal) staining of growth plate under indicated time points. GP, growth plate. Scale bars, 25 μm. **d** Immunofluorescence staining for IHH at E18.5, P14, 8w, 6m. Scale bars, 100 μm. The error bar represents the standard deviation of the mean. ns, no statistical significance, ****P* < 0.001.

**
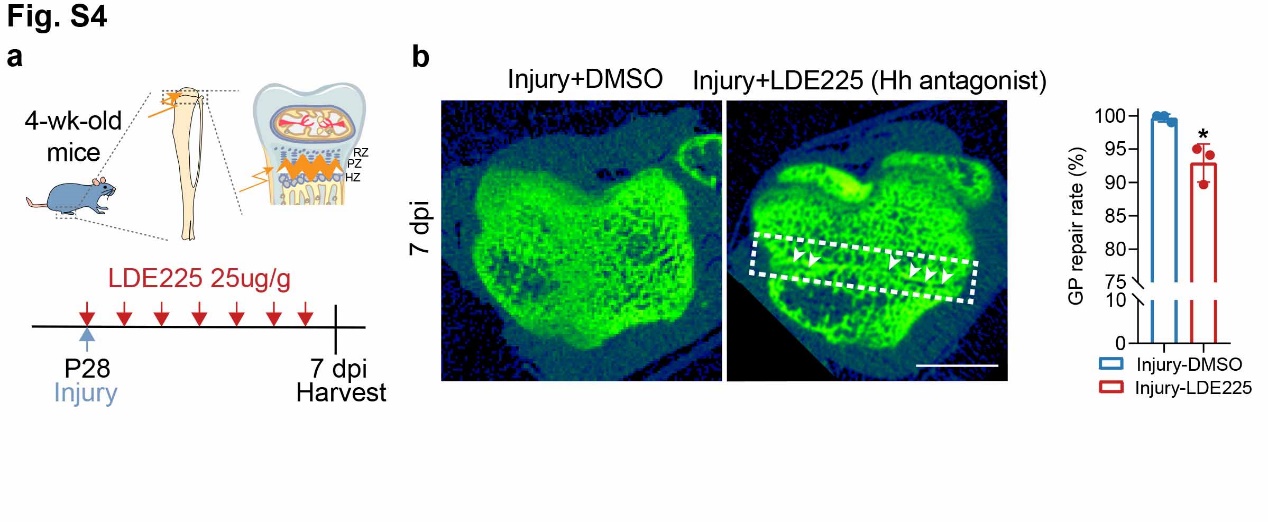
Fig. S4 Hedgehog signaling inhibition impairs GP repair. a** Schematics of injury model in the tibia growth plate with Hh antagonist (LDE225) administration. **b** Color maps of representative micro-CT images and quantification of operated tibias treated with DMSO or LDE225 at 7 dpi. White arrows indicate the injury gap. Scale bar, 1mm. n=3. GP repair rate = (gap volume at 1 dpi - gap volume at 7 dpi) / (gap volume at 1 dpi). The error bar represents the standard deviation of the mean. **P* < 0.05.

**
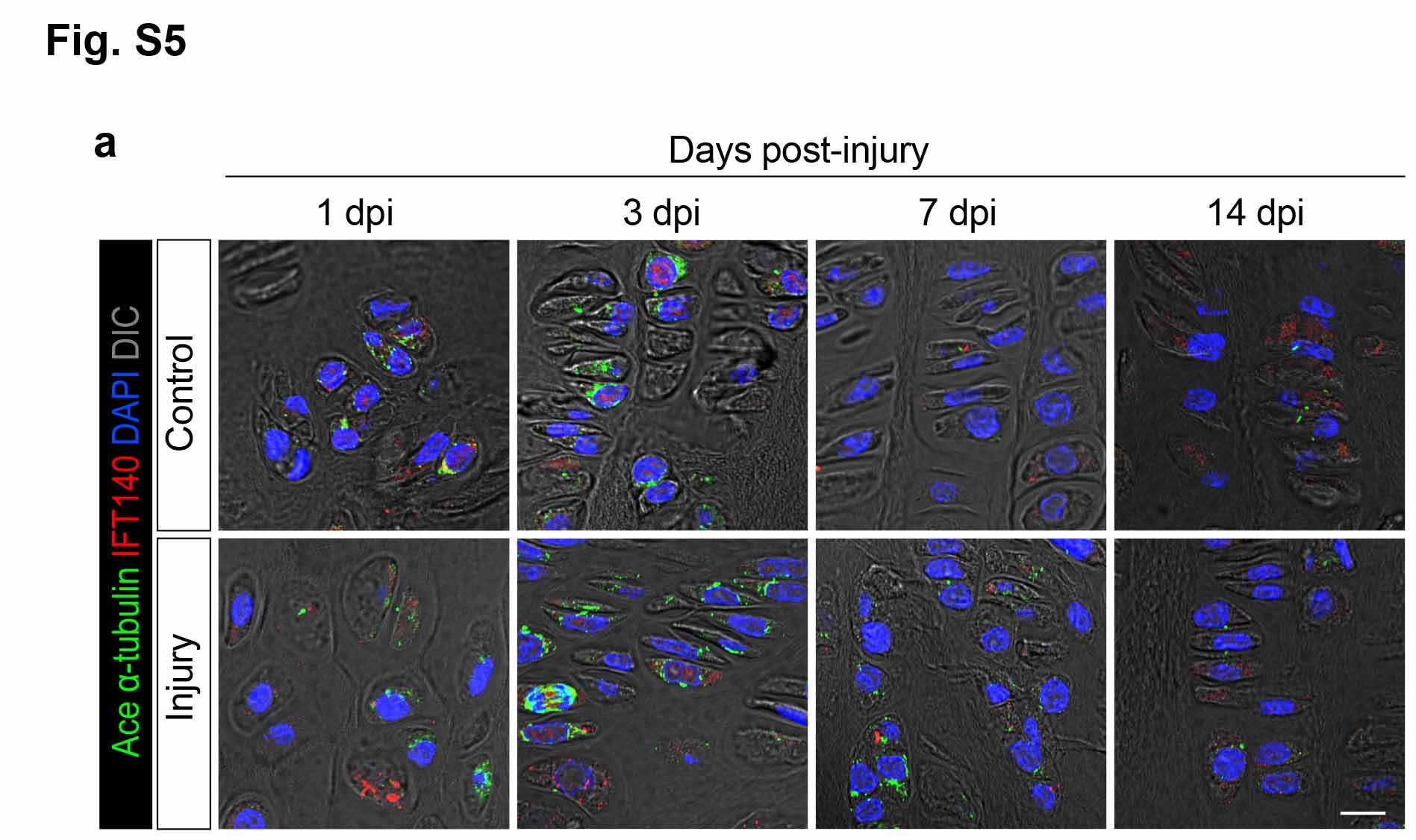
**

**Fig. S5** **Expression of IFT140 in growth plate during regeneration. a** Immunofluorescence staining for IFT140 of control and operated tibias at 1, 3, 7, and 14 days after surgery. Scale bars, 100 μm.
