# Supplementary material for: Primary cilia support cartilage regeneration after injury
Source: Int J Oral Sci. 2023 Jun 2;15:22. doi: 10.1038/s41368-023-00223-6 (PMC10238430; doi:10.1038/s41368-023-00223-6)
Supplement: Supplementary file 2 — Supplementary table [file 41368_2023_223_MOESM2_ESM.docx]

**Supplementary Table 1 Primer sequences**

| Primers | Forward 5’-3’ | Reverse 5’-3’ |
| --- | --- | --- |
| *Gapdh* | TGTGTCCGTCGTGGATCTGA | CCTGCTTCACCACCTTCTTGA |
| *Gli1* | CCAAGCCAACTTTATGTCAGGG | AGCCCGCTTCTTTGTTAATTTGA |
| *Ptch1* | AAAGAACTGCGGCAAGTTTTTG | CTTCTCCTATCTTCTGACGGGT |
